# Treatment effects of palliative care consultation and patient contentment: A monocentric observational study

**DOI:** 10.1097/MD.0000000000024320

**Published:** 2021-03-26

**Authors:** Lilit Flöther, Barabara Pötzsch, Maria Jung, Robert Jung, Michael Bucher, André Glowka, Daniel Medenwald

**Affiliations:** a Klinik für Anästhesiologie und Operative Intensivmedizin, Universitätsklinik und Poliklinik für Strahlentherapie; b Institut für Medizinische Epidemiologie, Biometrie und Informatik Universitätsmedizin Halle (Saale), Germany.

**Keywords:** FAMCARE-6, M.D. Anderson symptom inventory, palliative care consultation, symptom burden

## Abstract

Palliative care is a central component of the therapy in terminally ill patients. During treatment in non-palliative departments this can be realized by consultation.

To analyze the change in symptom burden during palliative care consultation.

In this observational study, we enrolled all cancer cases (n = 163) receiving inpatient treatment for 2015 to 2018 at our institution. We used the MDASI-questionnaire (0 = ‘not present’ and 10 = ”as bad as you can imagine”) and the FAMCARE-6 (1 = very satisfied, 5 = very dissatisfied) to analyze the treatment effect and patient satisfaction, respectively.

We examined the association of symptom burden and patient satisfaction using Spearman-correlation. Comparing mean values, we applied the Wilcoxon-test and one-way ANOVA.

An improvement in MDASI-core-items after treatment completion was significant (*P* < .05) in 14/18 symptoms. The change in perception of pain showed the strongest improvement (median: 5 to 3). Initially the MDASI-items “activity” (median = 8) and emotional distress (median = 5 and 6) were viewed as especially incriminating. There was no evidence for a correlation between patients’ age, the type of diagnosis and time since diagnosis.

The analysis of FAMCARE-6 patient contentment was lower or equal to two in all of the six items. There was a weak negative association between the change in symptom burden of psycho-emotional items “distress/feeling upset” (*P* = .006, *r*_Sp_ = −0,226), “sadness” and patient satisfaction in FAMCARE-6.

A considerable improvement of the extensive symptom burden particularly of pain relief was achieved by integrating palliative consultation in clinical practice.

## Abstract 2

Supplemental Digital Content is available in the text

## 1 Introduction

Following cardiovascular diseases, malignant disorders were the second most common cause of death in Germany in recent years.^[1]^ Against this background, the increasing importance of palliative medicine becomes evident. Palliative care focuses on a holistic and multi-professional support of terminally ill patients with a special focus on mental well-being.^[2]^

Hospitals with integrated specialized inpatient palliative care units or with palliative consultation service, where the patient is treated in a non-palliative department, cover inpatient palliative care in Germany.^[3,4]^ However, Erlenwein et al showed that in 2012 only 27% of German acute-care hospitals offer palliative care consultation of which university hospitals account for 58%.^[5]^ Palliative care consultation offers support through early palliative care integration for patients in advanced stages of an underlying disease. Palliative care consultation can improve the quality of life and satisfaction of patients and relatives during hospitalization and acute treatment in a specialized department.^[6,7]^

A prospective monocentric study by Hanson et al compared the change in symptom intensity and treatment costs in 304 patients. The authors compared two treatment groups with and without palliative care consultation. The study showed that palliative care cases had lower treatment costs, caused lower costs per day and showed stronger improvement in symptom intensity. The symptoms “pain,” followed by “shortness of breath,” and “nausea”^[8]^ improved strongest. However, limitations of this study are the short observation period of three days, as well as the extent of only five evaluated symptoms for assessing symptom burden. In addition, a third party with predetermined scores assessed the intensity of symptoms. Thus, a possible misjudgement of a subjectively perceived burden of the patient may lead to biased results.

Retrospective studies regarding the implementation of palliative care consultation in inpatient treatment showed a positive effect on incidental costs for laboratory tests and procedures. In the same vein, the number of admissions to the intensive care unit dropped.^[9,10]^ Regarding the outpatient integration of palliative care, data are more plentiful. A Norwegian study showed that interventions by a multi-professional palliative care team had advantages over conventional treatment without palliative care in terms of a death at home. A larger proportion of patients in the intervention group than controls died at home, while the latter died more frequently in hospitals. However, more patients of intervention group (receiving palliative care support) were admitted for inpatient treatment during their final months of life.^[11]^ A significant reduction in depressive state of mind and improved quality of life were advantages of the involvement of a palliative care team in overall care.^[12,13]^ Furthermore Temel et al^[12]^ showed a low usage rate of aggressive therapeutic strategies in palliative care (33% intervention, 54% control).

The objective of this study is to analyze the effect of palliative care consultation by assessing the change in symptom burden during palliative care support in cancer patients. Furthermore, we analyzed the association between changes in symptom burden and patient satisfaction. To the best of our knowledge, there are no recent studies, which describe the effect of palliative care consultation with an extensive survey of symptom burden in an inpatient every-day setting.

## 2 Materials and methods

### 2.1 Study sample

The presented work is a retrospective monocentric observational study conducted at the University Hospital Halle (Saale) and encompassed 163 cancer cases.

We included all adult inpatient cases (>18 years) who were hospitalized between September 2015 and August 2018 and were treated by the palliative service after consultation in addition to the primary oncological treatment. In order to avoid biases by selection, all patients were eligible to this study

The study was approved by the Ethics Committee of the University Medical School of the Martin Luther University Halle-Wittenberg on November 22, 2018 (Reference: 2018-114).

### 2.2 Data collection

The data were collected from the documentation of the basic palliative care assessment (PBA), which consisted of the core data set of the national hospice and palliative care register (HOPE), the self-assessment questionnaires Brief Pain Inventory,^[14,15]^

M.D. Anderson Symptom Inventory (MDASI),^[16]^ NCCN Distress Thermometer,^[17]^ and Hospital Anxiety and Depression Scale,^[18,19]^ and as third-party questionnaires the ECOG^[20,21]^ and Karnofsky Index.^[22]^

Patient satisfaction was measured by the FAMCARE-6 questionnaire after completion of palliative care. It is one of the internationally recognized and validated tools for measuring patient and family satisfaction.^[23]^

The MADSI questionnaire as a self-assessment questionnaire contains 19 items, whose presence and severity of symptoms within the past 24 h are assessed by the interviewed patients on a scale from 0 (no impairment) to 10 (severe impairment).^[16,24–26]^

The FAMCARE as a standardised tool with 20 items measures the satisfaction of patients and their relatives with the care provided by doctors and nursing staff.^[27]^ The short form FAMCARE-6 with six items^[23]^ includes the four original subscales “information transfer,” “accessibility,” “symptom control,” and “psycho-emotional care” (categories: very satisfied = 1, satisfied = 2, undecided = 3, dissatisfied = 4, very dissatisfied = 5). The survey of patient satisfaction was conducted during the final phase of palliative consultation.

### 2.3 Statistical methods

At the beginning, the data characteristics were described using descriptive statistics. Frequencies were tested for statistical significance by using a Chi^2^ test and metric variables by the nonparametric Wilcoxon sign test. We applied a single factor ANOVA and paired *t* tests (two-sided) to compare mean change between groups. We computed correlations between variables by using the non-parametric Spearman test. Cases with missing data were excluded from the analysis as there were few observations with missing data (Supplemental Digital Content: Table S1, http://links.lww.com/MD/F797).

FAMCARE 6 was evaluated both item-specifically and as the sum (sum of six variables with six categories = maximum value of 36) of the individual questions as an expression of overall satisfaction. We computed medians with the scattering measures of the 25th percentile and 75th percentile.

The significance threshold was set at *P* < .05 for all statistical analyses performed in this study.

Statistical analyses were performed with IBM SPSS Version 25.

## 3 Results

### 3.1 Patient characteristics

The investigated patient population included 163 palliative cases (70 male patients (=42.9%), 93 female patients (=57.1%). The predominant age of the population ranged between 50 and 79 years (79.8%), while patients between 50 and 59 years formed the largest group.

Coming to tumor entities, lung cancer was the most frequent cancer type with a number of 48 patients (=29.4%), followed by the group of malignant gastrointestinal tumors (n = 34, 20.9%).

The predominant patient subgroup with 53 patients (=33.3%) was the one with a maximum period of 12 months since diagnosis, followed by the subgroup of newly cancer diagnoses (≤8 weeks) with 49 patients (=30.8%). Coming to tumor entities, most relevantly 24 (=50.0%) of 48 lung cancer patients were newly diagnosed carcinomas (time since diagnosis fewer than 12 months), while in 16 patients (=33.3%) the time since diagnosis exceeded 12 months. With 67 of 163 patients (=41.1%), the subgroup with a length of stay longer than 21 days was most frequently represented (Table 1).

**Table 1:** Characteristics of the study population.

Study samples		
Number	N = 163	
Gender		
Male	70/163	42.9%
Female	93/163	57.1%
Age		
<50 years	9/163	5.5%
50–59 years	50/163	30.7%
60–69 years	35/163	21.5%
70–79 years	45/163	27.6%
>80 years	24/163	14.7%
Diagnosis		
Gynecological tumors	27/163	16.6%
Urological tumors	16/163	9.8%
Hematological tumors	5/163	3.1%
Gastrointestinal tumors	34/163	20.9%
Lung tumors	48/163	29.4%
Other tumor disease	28/163	17.2%
No tumor disease	5/163	3.1%
Time since recognition		
Since 8 weeks	49/159	30.8%
Since 12 months	53/159	33.3%
Since 2 years	19/159	11.9%
Since 5 years	21/159	13.2%
>5 years	17/159	10.7%
Duration of stay in hospital		
1–7 days	6/163	3.7%
8–14 days	41/163	25.2%
15–21 days	49/163	30.1%
>21 days	67/163	41.1%
NCCN distress	Mean (Std.err.)	
Anxiety (HADS)	8.9	0.3
Depression (HADS)	10.1	0.3
NCCN distress	6.4	0.1
ECOG (start)	3	(3–4)
Karnofsky (start)	45	(30–50)
ECOG (end)	3	(3–4)
Karnofsky (end)	50	(30–50)

ECOG = Eastern Co-operative of Oncology Group, HADS = Hospital Anxiety and Depression Scale.

There was no significant change in both, the ECOG (*P* = .52) and Karnofsky index (*P* = .06) when the beginning and the end of treatment were compared (Table 1).

**Figure 1:**
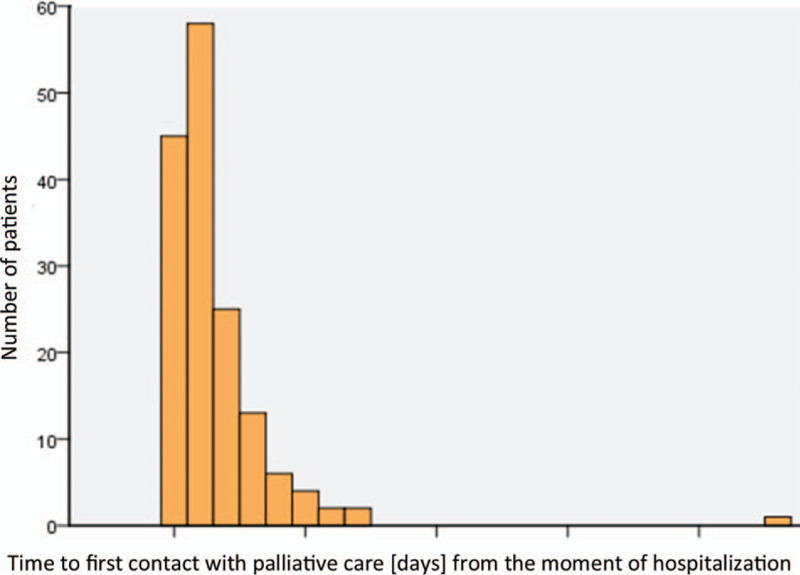
Distribution of time to first contact with palliative care after hospitalization.

The analysis of the time of entry into palliative care showed that 24 patients (=15.4%) were enrolled in the palliative service within 1 day and 17 patients (=10.9%) within 2 days after hospital admission (Fig. 1).

### 3.2 MDASI

The analysis of the MDASI-questionnaire showed that the patients reported impairments especially in three large symptom groups at the beginning of the palliative complex treatment.

Most importantly, the category of physical activity with its items of ”everyday activity“ ($\bar{x}$ = 8, IQR 7-9) and ”walking ability“ ($\bar{x}$ = 8, IQR 5-10) showed the highest values across all categories.

**Figure 2:**
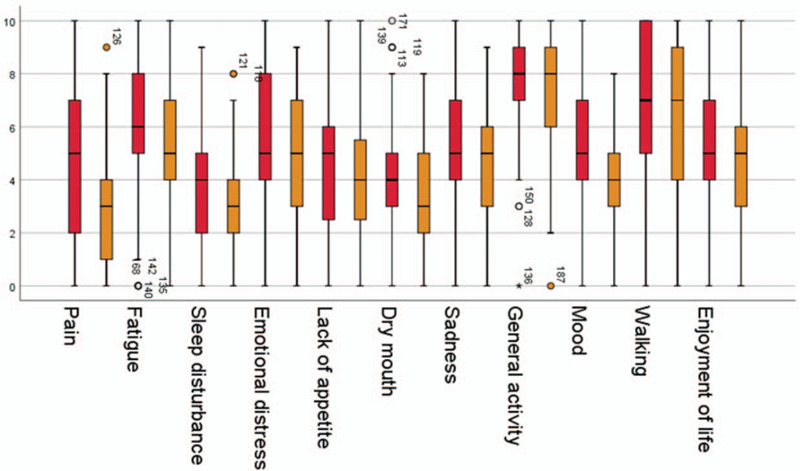
Comparison of MDASI-Items at the beginning and end of treatment (red: beginning, orange: end of treatment).

The items of psychoemotional stress (”joie de vivre“ [$\bar{x}$ = 5, IQR 4–7], ”mood“ [$\bar{x}$ = 5, IQR 4–7], ”sadness“ [$\bar{x}$ = 5, IQR 4–7], ”worries“ [$\bar{x}$ = 6, IQR 4.75–8] and ”fatigue“ [$\bar{x}$ = 6, IQR 5–8]) were found to be particularly impairing. Patients reported a medium pain level with a median value of $\bar{x}$ = 5 (IQR 2–7, Fig. 2).

After completion of the palliative co-management, 14 MDASI items achieved a significant improvement compared to the initial situation (Supplemental Digital Content: Table S2, http://links.lww.com/MD/F798). The symptom ”pain“ was especially prone to improvements with a decrease in the median from five to three. For seven MDASI-items the median could be reduced by one point. The items ”Distress,“ ”Sadness,“ and ”Enjoyment of life“ as well as ”Activity“ and ”Walking ability“ showed no improvement.

A correlation between an improvement in symptom load and age group, cancer types and time since diagnosis” could not be demonstrated (*P* > .05 for Chi^2^ significance test).

There was no evidence indicating that a particular subgroup showed a significantly different effect from other subgroups (Supplemental Digital Content: Table S3, http://links.lww.com/MD/F799 of the appendix).

### 3.3 FAMCARE-6

**Figure 3:**
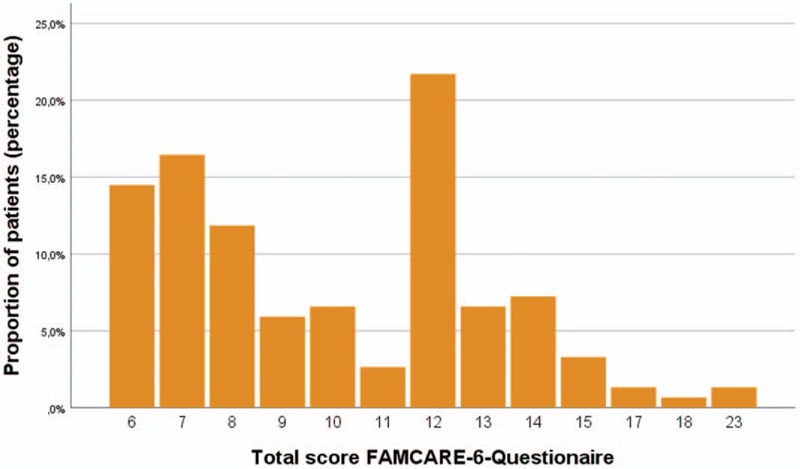
Distribution of FAMCARE-6-total score as patient satisfaction.

Figure 3 shows the distribution of FAMCARE total scores among patients.

The analysis of the item change in comparison to the total score achieved in FAMCARE-6 showed a weak correlation between patient satisfaction and the improvement of the MDASI items “worries” (correlation after Spearman-Rho = −0.226, *P* = .006;) and “sadness” (correlation after Spearman-Rho = −0.206), *P* = .012. The smaller the change in symptom burden in MDASI, the higher the dissatisfaction, expressed by a higher total sum in FAMCARE-6.

A correlation between the items “pain,” “sleep disorders,” “loss of appetite,” “dry mouth,” “mood,” and “joy of life” (correlation according to Spearman-Rho = −0.154 and 0.102, *P* > .05 results not shown) could not be shown, nor was there any significant correlation between age groups, time since diagnosis and tumor type and the total sum in FAMCARE-6. (*P* > .05; ANOVA univariate).

## 4 Discussion

The analysis of the initial symptoms in the study population shows that cancer patients entered complex palliative care in an already significantly reduced general condition with considerable limitations in self-care and significant psychoemotional stress. Palliative patients with moderate symptom load were hardly represented in this oncological setting.

A cancer diagnosis is a severe psychological burden for patients.^[28]^ If there is a lack of compensatory capacity due to reorientation or rapid progression of the disease, the adaptation disorder may become chronic. This applies to one third of the patients with malignant primary disease.^[29]^ It manifests itself, among other things, in the form of depression, anxiety disorders, mixed affective states or as a severe form of tumor-related fatigue syndrome.^[29,30]^

Unfortunately, these psychologically severely impaired patients are currently hardly recognised in medical care and therefore inadequately treated, which has negative consequences for the length of hospital stay, therapy motivation, and the course of treatment.^[30]^

This study revealed a significant improvement of the complex symptom load during palliative medical treatment after consultation when embedded in primary treatment.

It is also important to ensure constant psycho-oncological monitoring throughout the course of the disease.^[29]^

The presence of lung cancer patients with significantly restricted living conditions was dominant. Due to the lack of early detection options and unspecific symptom burden over a long period of time in the development of this disease pattern such adverse conditions are aggravated.^[31,32]^

However, in our study we used the MDASI rather than the specific version for lung cancer patients MDASI-LC to make effect comparable. The latter includes the items “coughing,” “swallowing disorders,” and “constipation,” which have been frequently detected in patients with malignant basic lung diseases.^[26]^ This might lead to slightly altered changes in recorded symptoms.

In this study, the FAMCARE-6 was used to survey patient satisfaction in the inpatient setting. After analysis of the results, this questionnaire appears to differentiate little. Thus, it might be of restricted value for the use in the inpatient setting.

This is suggested by the remarkably high assessment of satisfaction with only little variance for a rather small size of the study population. It should be mentioned that the interviewees did not differentiate between the individual departments and thus no conclusion could be drawn about a particular care discipline.

FAMCARE was originally developed for the care of palliative patients in an outpatient setting, in which knowledge about side effects and the availability of specialist staff play a much greater role, since both care and the assessment of changes in the situation are the responsibility of the caring family member. In contrast, there is the inpatient setting, where medical personal can be provided in sufficient numbers for the most part.^[23]^

Likewise, the important sub-area of communication does not appear to be adequately represented in FAMCARE-6.

A patient-centered and empathic dialogue between patients and the treating staff is an important factor in medical care. As problems are better recognized, the understanding of the patients about the clinical picture, symptoms, and existing therapy options and thus compliance is increased which is associated with a better outcome. Therefore, this should be taken into account when measuring patient satisfaction.^[33,34]^ As our results indicate this can be fulfilled by consultation during primary treatment.

In the light of a more and more complex cancer treatment, it appears mandatory to combine disciplines in order to elevate symptom burden. This becomes especially evident, as the survival prospect of patients in a metastasized stage has improved during recent years.^[6,7,12]^ Thus, palliative care consultation can combine the advantages of primary cancer treatment with supportive care and symptom control. Finally, patients might endure treatment better and thus cancer treatment by chemo- or immunotherapy without losing the benefit of palliative care.

One limitation of this study is the lack of a comparative population of palliative patients without treatment by the palliative service. Thus, it could not be proven beyond doubt which part of the symptom burden improvement is attributable to the palliative service and which to the primary care clinic. However, it would be unethical to withhold palliative care from cancer patients in an advanced stage of their disease. Thus, we can only assume that the condition of the patients would not have changed had they not received palliative care. This reasoning is supported by the fact that patients in our study had a long history of their disease.

Our results refer to a single institution, but due to the broad inclusion criteria can be generalized to a wide variety of clinics with palliative care consultation. This is especially true as the patient cohort was not limited to a particular entity.

This scientific work was based on the MDASI questionnaire, from which a large part of the symptom burden was collected in a structured survey. This questionnaire is not an obligatory tool for the anamnesis survey within the different departments. By using different tools, a retrospective comparability of the complex symptom burden and its dynamics during the inpatient stay between patient groups with and without palliative care would be insufficiently assessable.

## 5 Conclusions

The implementation of a palliative medical consultation service in inpatient care for palliative patients with an advanced underlying disease enables early care in the sense of the Early Palliative Care Model even during specialized treatment of the underlying disease.

This study was able to show that palliative medical consultations resulted in a significant improvement of the extensive symptom burden, especially in the extent of pain symptoms, which were reduced the most. After the items for physical impairment, the burden of symptoms of the psychoemotional items was highest during the first contact with palliative care. In this study, a reduction in psychoemotional stress correlated with higher patient satisfaction.

## Author contributions

**Conceptualization:** Daniel Medenwald.

**Data curation:** Barabara Pötzsch, Daniel Medenwald.

**Formal analysis:** Barabara Pötzsch, Maria Jung, Daniel Medenwald.

**Methodology:** Daniel Medenwald.

**Project administration:** Lilit Flöther, Daniel Medenwald.

**Supervision:** Lilit Flöther, Michael Bucher, André Glowka, Daniel Medenwald.

**Validation:** Michael Bucher, André Glowka.

**Writing – original draft:** Robert Jung, André Glowka, Daniel Medenwald.

**Writing – review & editing:** Maria Jung, Robert Jung, André Glowka, Daniel Medenwald.
